# Biochemical composition and antioxidant activity of three extra virgin olive oils from the Irpinia Province, Southern Italy

**DOI:** 10.1002/fsn3.1180

**Published:** 2019-09-06

**Authors:** Florinda Fratianni, Rosaria Cozzolino, Antonella Martignetti, Livia Malorni, Antonio d’Acierno, Vincenzo De Feo, Adriano G. da Cruz, Filomena Nazzaro

**Affiliations:** ^1^ Istituto di Scienze dell’Alimentazione Consiglio Nazionale delle Ricerche (CNR‐ISA) Avellino Italy; ^2^ Dipartimento di Farmacia Università degli Studi di Salerno Fisciano Italy; ^3^ Departamento de Alimentos, Instituto Federal de Educação Ciencia e Tecnologia de Rio de Janeiro (IFRJ) Rio de Janeiro Brazil

**Keywords:** antioxidant activity, extra virgin olive oil, polyphenols, statistical analysis, volatile compounds

## Abstract

Extra virgin olive oil (EVOO), appraised for its healthy properties, represents an important element for the economy of several countries of the Mediterranean area, including Italy. Our study aimed to evaluate some biochemical characteristics (polyphenols and volatile compounds) as well as the antioxidant activity of three EVOOs obtained from the varieties *Ravece*, *Ogliarola,* and *Ruvea antica*, grown in the same field of an Irpinian village, Montella, in the Campania region, Southern Italy. Extra virgin olive oil *Ruvea antica* contained the greatest amount of total polyphenols and showed the highest antioxidant activity. Principal component analysis of the aromatic profiles indicated that the three EVOOs could be easily discriminated according to the cultivar. 1‐Hexanol, 2‐hexen‐1‐ol, 3‐pentanone, representing the most abundant volatiles of the EVOO *Ruvea antica*, and 2‐hexenal, which resulted the main component in EVOOs *Ogliarola* and *Ravece*, could be considered as markers to discriminate these three EVOOs, according to the ReliefF feature selection algorithm.

## 1 INTRODUCTION

Extra virgin olive oil (EVOO) is the most precious product obtained by the milling of the fruits of the olive tree (*Olea europaea* L. subsp. *europaea* var. *europaea*), one of the most worldwide cultivated plants (Antolini, 1997). At present, about 1,500 cultivars and/or varieties are known, as well as 3,000 synonyms of registered cultivars, ecotypes, and local varieties, which overall contribute to the enormous capital of olive germplasm. Italy, Spain, France, Tunisia, and Greece have the highest number of cultivars and varieties (Doveri & Baldoni, 2007). Extra virgin olive oil can be considered as a “lipid fruit juice” obtained from the fresh fruits of the olive by physical and mechanical processes. The strong link between the cultivar and the territory of cultivation makes it a product with particular characteristics, especially from the point of view of organoleptic, nutritional, and healthy properties. The EVOO plays a key role in the eating habits of the Mediterranean peoples, and since ancient time, it is very popular in several dietary regimes, and still today it represents a pillar of the Mediterranean diet. The variety/cultivar, climatic conditions of the cultivation site, agronomic practices, degree of ripening, storage conditions, and techniques of fruit processing are all factors that may affect the quality and the sensorial characteristics of EVOO, as well as its biological properties (Lazzez, Perri, Caravita, Khlif, & Cossentini, 2008). The variety certainly represents one of the most important factors in determining the quality of the oil, which can vary greatly also for genetic reasons (Tura, Failla, Bassi, Pedo, & and, 2008). However, olives of the same variety, cultivated under different environmental conditions or in diverse geographical areas, can produce EVOOs with different organoleptic characteristics and healthy properties (Angerosa, Basti, Vito, & Lanza, 1999). Concurrently, fruits from different cultivars grown under the same environmental conditions could produce oils with different biochemical characteristics (Gorzynik‐Debicka et al., 2018). In the composition of EVOOs, volatile organic compounds (VOCs) and polyphenols are of great importance. Volatile organic compounds are strongly related to oil aroma perceived during the assay of the product (Salas, Harwood, & Martinez‐Force, 2013). They are produced at the beginning of the malaxation, during cell structure rupture, due to enzymatic reactions in the presence of oxygen. C6 aldehydes, C6 alcohols, and their corresponding esters, together with smaller amounts of C5 carbonyl compounds, are the main constituents of VOCs (60%–80%). Specifically, hexan‐1‐ol, hexanal, E‐2‐hexenal, and 3‐methylbutan‐1‐ol generally dominate the VOCs pattern of the most common EVOOs from Mediterranean regions (Salas et al., 2013). volatile organic compounds profile can depend on cultivar and on degree of maturation (Angerosa et al., 1999).

The Mediterranean diet is the golden standard for healthy nutrition. It is characterized mainly by a high intake of fruit, vegetables, and cereals, which are rich in phytochemicals (Fratianni et al., 2016). Among these compounds, polyphenols stimulated particular attention, due to their versatility of action, being able to protect against oxidative stress and to inhibit the proliferation of cancer cells (Del Rio, Costa, Lean, & Crozier, 2010). The beneficial effects of the Mediterranean diet are also attributed to the EVOO (Visioli & Bernardini, 2011), which, even if more expensive than olive oil, is richer in polyphenols, vitamins, phytosterols, etc., concurring to reduce the risk of cardiovascular events (Estruch et al., 2013), so that US Food and Drug Administration compared it to a real drug. Extra virgin olive oil is rich in polyphenols ranging between 50 and 1,000 mg gallic acid equivalents (GAE)/kg of product (Gorzynik‐Debicka et al., 2018). Oleuropein, quercetin, and hydroxytyrosol, some of the main polyphenols present in EVOO, have antioxidant activity and ascertained effects in protecting against the coronary artery disease (Manna et al., 2002) or cancer (Owen et al., 2000).

The aim of our work was to determine the biochemical composition of three EVOOs obtained from traditional varieties of olives cultivated in the same field of Montella, a little village of the Irpinia region, Southern Italy, harvested in the same period and processed by cold pressure. Three varieties, Ogliarola, Ravece, and Ruvea antica, in particular, attracted our attention. These are typical varieties of the Mediterranean area, diffused in Campania. Tree of Ogliarola has a medium foliage, with elliptical‐lanceolate leaves. It produces a low number of flowers. Its fruits are black and elliptical, with a weight of 2–4 g. The endocarp has a weight of 0.3–0.45 g. Ravece tree has a high foliage density. Leaves are elliptical‐lanceolate. Fruits are elongated, purple, and have a weight of 4–6 g; the endocarp is heavy (weight >0.45 g). Ruvea antica tree has medium foliage. Leaves are elliptical‐lanceolate and longer more than 7 cm. Its fruits are purple, elliptical, and show a weight of 2–4 g. The endocarp has a weight of 0.3–0.45 g (Di Vaio & Nocerino, 2012). The biochemical characterization of resulting EVOOs involved the total antioxidant activity and the polyphenol content. The polyphenolic profile and VOCs were also evaluated. Statistical analysis allowed us to correlate some of the biochemical characteristics of the EVOOs; in particular, the antioxidant activity was correlated with total polyphenols and the singular components, identified in the oil by UPLC. Principal component analysis (PCA) of the aromatic profiles (obtained by Gas Chromatography/Mass Spectrometry) was carried out to discriminate oil samples according to cultivar. Moreover, a feature selection algorithm was used to identify and select putative volatile markers responsible for EVOO varieties discrimination.

## 2 MATERIALS AND METHODS

### 2.1 Materials

Caffeic, ferulic, *p*‐coumaric, gallic, and chlorogenic acids, catechin, quercetin, 3‐hydroxytyrosol, spiraeoside, oleuropein, daidzein, luteolin, naringenin, formononetin, 2,2‐diphenyl‐1‐picrylhydrazyl (DPPH), HPLC‐grade ethanol, 4‐methyl‐2‐pentanol, and acetonitrile were purchased from Sigma‐Aldrich. Apigenin and hyperoside were purchased from Extrasynthese. Ethanol was obtained from Romil. Ultrapure water from a Milli‐Q system (Millipore) with a resistivity at 25°C of 18 MΩ ∗ cm was used throughout the analyses. Helium (Rivoira) at a purity of 99.999% was the GC carrier gas. The SPME glass vials and the fibers were from Supelco; the capillary GC‐MS column HP‐Innowax (30 m × 0.25 mm × 0.5 μm) was purchased from Agilent J&W (Agilent Technologies Inc.).

### 2.2 Plant material

The EVOOs used in this study were produced in the same year by cold pressing of three different varieties (*Ruvea antica*, *Ogliarola,* and *Ravece*) grown in the same field located in the Montella village, in the Irpinia Province, Campania region, Southern Italy. Prof. Vincenzo De Feo identified the varieties. Voucher specimens of the three varieties were stored in the herbarium of the Department of Pharmacy, University of Salerno.

### 2.3 Polyphenol analysis and free radical scavenging capacity

To isolate the phenolic fraction of the three EVOOs, 1.5 g of sample was mixed with 1.5 ml of hexane and charged onto cartridges SPE C_18_. Polyphenols were eluted through 3 ml of methanol 100% and recovered; this step was repeated other two times. The three residues were collected, grouped, dried, and re‐suspended with 1 ml of methanol. The samples were filtered (mesh = 0.20 μm). The method of Singleton and Rossi (Singleton & Rossi, 1965) was used to evaluate the content of total polyphenols present in the three EVOO samples. Quantification was determined by using gallic acid as standard and reading the absorbance at 760 nm through a Cary UV/Vis spectrophotometer (Varian). Results were expressed as μg gallic acid equivalent (GAE)/g of EVOO ± standard deviation (*SD*).

The free radical scavenging activity was determined using the stable radical 2,2‐diphenyl‐1‐picrylhydrazyl (DPPH assay) (Brand‐Williams, Cuvelier, & Berset, 1995). The analysis was performed in microplates by adding 15 μl of extract to 300 μl of a methanol DPPH solution (0.153 M). Next, the absorbance at *λ* = 517 nm was spectrophotometrically measured (Cary 50 MPR, Varian). The absorbance of DPPH without antioxidant (control sample) was used for baseline measurements. The scavenging activity was expressed as effectiveness (%) of the sample to inhibit DPPH radical activity during a 60‐min incubation.

Polyphenol profile was determined through UPLC (ultra high‐performance liquid chromatography) by using an ACQUITY Ultra Performance system linked to a PDA 2996 photodiode array detector (Waters), setting the UV detection wavelength at 280 nm, following the method of Fratianni and coworkers (Fratianni et al., 2016). Quantification of known components was performed by comparing the peak areas on the chromatograms of samples with those obtained from standard solutions.

### 2.4 Analysis of VOCs profiles

The optimization of SPME parameters was achieved by examining samples of a commercial EVOO bought at a local supermarket. SPME GC‐MS volatile analysis was accomplished according to Romero and coworkers (Romero, Garcıa‐Gonzalez, Aparicio‐Ruiz, & Morales, 2015), but using the DVB/CAR/PDMS (50/30 μm) fiber. For the sample preparation, 2 g of each sample was put into a 20‐mL headspace vial with screw cap (Supelco) and 4‐methyl‐2‐pentanol to a final concentration of 1.5 mg/g was added as an internal standard to guarantee the analytical reproducibility. Subsequently, vials, closed with a Teflon (PTFE) septum and an aluminum cap (Chromacol) and stirred, were put in the instrument dry block heater and held at 40°C for 10 min. After the equilibration time, the extraction and injection processes were automatically carried out using an autosampler MPS 2 (Gerstel).

Volatiles were analyzed by gas chromatography‐quadrupole mass spectrometry (GC‐qMS), introducing the SPME fiber into the injector port of the gas chromatographer, model GC 7890A, Agilent hyphenated with a mass spectrometer 5975C. Once desorbed, metabolites were directly transferred to the capillary column HP‐Innowax for the analysis. The oven temperature program was initially set at 40°C for 3 min, increased to 200°C at 30°C/min, and then ramped to 240°C at 30°C/min, holding for 1 min. Volatiles were investigated according to the instrumental parameters as reported in the literature (Cozzolino, Martignetti, et al., 2016; Cozzolino, Pace, et al., 2016). Each sample was analyzed in duplicate in a randomized sequence where blanks were also run. Volatile metabolites recorded in the headspace of the extra virgin olive oils under study were identified by three diverse methods, as previously reported (Cozzolino, Martignetti, et al., 2016; Cozzolino, Pace, et al., 2016). The areas of the identified volatiles were determined from the total ion current (TIC), and the semiquantitative data of each metabolite (Relative Peak Area, RPA%) were considered in relation to the area of the peak of 4‐methyl‐2‐pentanol, used as internal standard.

### 2.5 Statistical analysis

Data were expressed as the mean ± standard deviation (*SD*) of triplicate measurements, and antioxidant activity was correlated with polyphenols. As concerns VOCs, analysis of variance (ANOVA) was used to compare results and significance was accepted at *p* < .05. Principal component analysis (PCA) was then used to relate the obtained values and as an explorative tool for the preliminary visualization of the separation of the different EVOO samples, according to their VOCs profiles. Last, the ReliefF (Kononenko, Simec, & Robnik‐Sikonja, 1997) feature selection algorithm was used to identify potential markers, among VOCs, responsible for EVOO discrimination.

## 3 RESULTS AND DISCUSSION

### 3.1 Total polyphenol content and antioxidant activity

**Figure 1:**
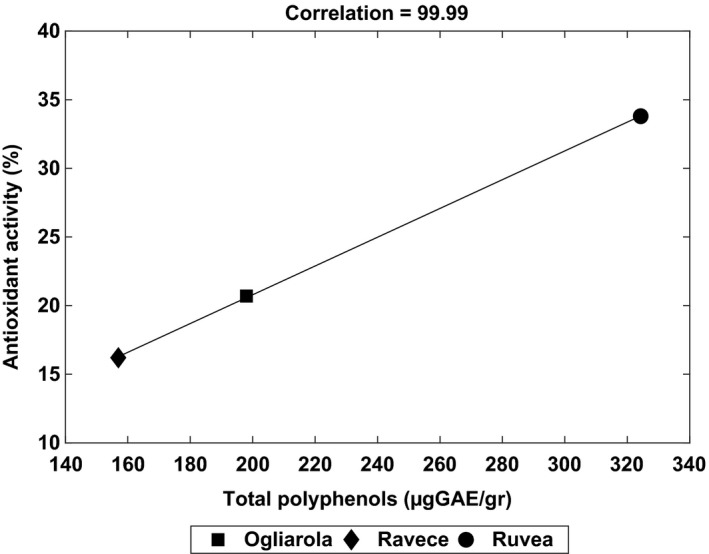
Correlation between total polyphenols found in the three EVOOs (expressed as μg GAE/g of product) and the antioxidant activity (calculated through the DPPH test and expressed as percentage)

The analysis of total polyphenols (TPF, Table 1) indicates that the amount of TPF present in the three EVOOs ranged between 156.96 and 324.27 μg GAE/g of EVOO. These values were coherent with the data of Di Giovacchino and coworkers (Di Giovacchino, Sestili, & Di Vincenzo, 2002). *Ruvea antica* and *Ogliarola* exhibited a content of TPF very close to that exhibited by some monovarietal Spanish EVOOS, such as “Cordoba” and “Sevilla” (Oliveras‐Ferraros et al., 2011). The antioxidant activity, evaluated through the DPPH test (whose results were expressed as percentage inhibition of DPPH, Table 1), indicated that the EVOO of *Ruvea antica* showed the best antioxidant activity (33.8%). Its efficacy was superior to the *Ogliarola* EVOO (20.7%) and mainly to the *Ravece* EVOO, which exhibited an effectiveness in inhibiting the DPPH radical even by half than *Ruvea antica*. The three EVOOS showed a perfect degree of correlation between the total polyphenol content and the antioxidant activity (corr = 99.99, Figure 1). A high correlation between polyphenols and antioxidant activity has been also demonstrated by Samaniego Sanchez and coworkers (Samaniego Sánchez et al., 2007).

**Table 1:** Total polyphenols (expressed as μg GAE/g of EVOO ± *SD*) and antioxidant activity (evaluated through the DPPH and expressed as percentage ± *SD*) of the three polyphenolic extracts from Ogliarola, Ravece, and Ruvea antica EVOOs

Variety	Total polyphenols		Antioxidant activity	
	μg GAE/g of EVOO	*SD*	%	*SD*
*Ogliarola*	198.5	±14.7	20.7	±2.227
*Ravece*	156.97	±12.91	16.2	±1.601
*Ruvea antica*	324.275	±6.91	33.8	±0.368

### 3.2 Polyphenol profile

The amount (expressed as μg GAE/g of EVOO) of polyphenols identified through UPLC analysis is shown in Table 2. In our study, we tried to eliminate as many variables as possible: For this reason, we took into consideration three olive cultivars grown in the same plot of land, collected, and processed in the same period using the same technology. The resulting EVOOs exhibited a quite varied polyphenols profile, both for the presence and the amount of constituents. Quercetin resulted very abundant in all samples. It ranged between 28.34 μg GAE/g of oil in *Ravece* and 47.65 μg GAE/g in *Ogliarola*, with an intermediate value (34.43 μg GAE/g) in *Ruvea antica*. Oleuropein, representing an abundant polyphenol in *Ogliarola* and *Ruvea antica*, was found at concentrations much lower in EVOO *Ravece* (9.30 μg GAE/g), the 5.93% of the total polyphenols. This molecule is an ester of hydroxytyrosol; it gives rise from the mevalonic acid pathway (Omar, 2010). Since an inverse relationship between fruit ripening and content of oleuropein occurs during the development of olive fruit (Kalua, Allen, Bedgood, Bishop, & Prenzler, 2005), the fruits of *Ravece*, though collected in the same period, were probably more mature respect to *Ogliarola* and mainly to *Ruvea antica*. This could be confirmed also by the presence/absence of the flavonoid apigenin, which was detected only in the polyphenolic extract of *Ruvea antica*: Taking into account that apigenin gives rise from naringenin, a variable degree of immaturity might be considered. Probably, the EVOO *Ruvea antica* was the most immature, conversely to EVOO *Ravece*, the most mature, with EVOO *Ogliarola* exhibiting an intermediate rate of maturity. Furthermore, taking into consideration the amount of oleuropein and naringenin, we might hypothesize that the EVOO *Ogliarola* could be closer, from the point of view of maturity, to *Ravece* rather than *Ruvea antica*. Such hypothesis is supported also by the total polyphenols content and the antioxidant activity of the EVOO *Ogliarola,* which values were more similar to those of the EVOO *Ravece* than to EVOO *Ruvea antica*. Spiraeoside (quercetin‐4′‐glucoside), a derivative of quercetin, was detected in similar amounts (18.78 and 18.67 μg GAE/g, respectively) in EVOOs *Ogliarola* and *Ruvea antica*. Concurrently, we found luteolin in *Ravece* and *Ruvea antica* (9.77 and 5.09 μg GAE/g, respectively), but not in *Ogliarola*. The polyphenols identified in the three EVOOs are well known highly bioavailable molecules. The presence of high amounts of oleuropein, whose absorption in the body is about 55%–60% (Omar, 2010), is very significant, given the numerous and key effects of such metabolite including antioxidant, anti‐inflammatory, anticancer, antiatherogenic activities, and cardioprotective, antihyschaemic and hypolipidemic properties (Visioli & Galli, 2002). Concomitantly, the high content of quercetin contributes to improve the biological value of the three EVOOs. The amount of quercetin and its derivative spiraeoside in the EVOOs *Ogliarola* and *Ruvea antica* represented the 51.74% and 35.04%, respectively, of the polyphenols. A so high amount of these compounds is certainly essential: Like other flavonoids, they can affect the cellular function, by mediating gene expression and signal transduction rather than through a direct antioxidant effect (Nemeth et al., 2003). Dietary quercetin and other flavonoids are absorbed by a little percentage (5%–10%) in the small intestine; the residue of these molecules moves to the colon, where they are metabolized by the gut microbiota, influencing its composition. These molecules exert potential prebiotic effect, protecting from intestinal dysbiosis and all alterations interesting microbiota, and finally, they can concur to significantly influence host biochemistry and host susceptibility to diseases (Nazzaro, Fratianni, d’Acierno, & Coppola, 2013; Tamura et al., 2017).

**Table 2:** Polyphenolic profile of the three EVOOs resulting from the UPLC analysis (expressed as μg GAE/g of EVOO)

Polyphenols	*Ogliarola*	*Ravece*	*Ruvea antica*
Gallic acid	0.00	0.00	0.00
3 Hydroxytyrosol	3.68	0.68	3.57
Catechin	2.13	0.00	1.40
p‐Coumaric acid	0.00	0.45	0.36
Quercetin‐4′‐glucoside (spiraeoside)	18.78	0.00	18.67
Oleuropein	31.23	9.30	41.58
Daidzein	8.18	0.00	7.66
Luteolin	0.00	9.77	5.09
Quercetin	47.65	28.34	34.43
Apigenin	0.00	0.00	10.34
Naringenin	7.90	10.31	21.05
Formononetin	8.82	7.55	7.37

**Figure 2:**
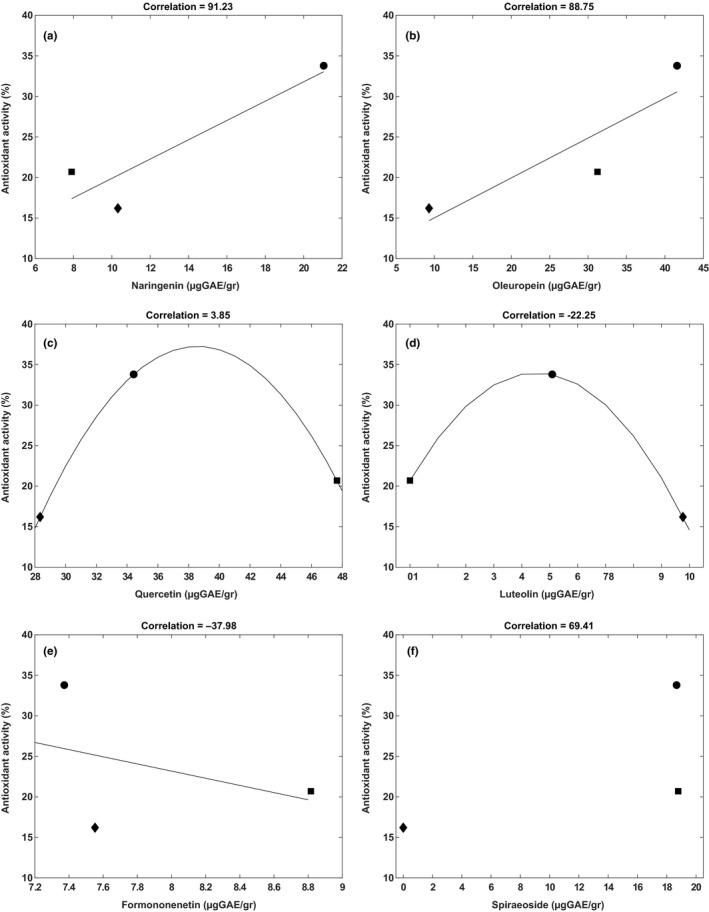
Correlation between the amount of some polyphenols with the antioxidant activity (calculated through the DPPH test and expressed as percentage) for *Ogliarola* (■), *Ravece* (♦), and *Ruvea antica* (●)

Considering the almost complete linearity between the total polyphenol content and the antioxidant activity (corr = 99.9, Figure 1), we used a statistical approach to evaluate the putative influence of some singular polyphenols on the antioxidant activity exhibited by the three extracts. This approach was previously used to study the influence of polyphenols on some biochemical characteristics and biological properties of different ecotypes of *Phaseolus vulgaris* L. (Ombra et al., 2016). The analysis was performed taking into consideration the most abundant molecules present in the three extracts, which resulted, by the UPLC analysis, quercetin, oleuropein, spiraeoside, formononetin, naringenin, and luteolin. The results are shown in Figure 2. Naringenin, present in all three polyphenolic extracts, at amounts ranging between 7.90 and 21.05 μg GAE/g EVOO, did not seem to affect the antioxidant activity in marked way until 10.31 μg GAE/g. Its effect seemed stronger upper such threshold, so that, at twice amounts, a doubling of the antioxidant activity was observed (Figure 2a). Oleuropein appeared to exhibit a linear behavior, with an antioxidant activity growing concurrently to its amounts (corr = 88.75, Figure 2b). This molecule seemed to be the main responsible for the antioxidant activity exhibited by the three polyphenol extracts, although it did not represent the most abundant molecule. Therefore, the noticeable antioxidant activity of oleuropein is reported, mainly as a scavenger of chain‐propagating lipid peroxyl radicals within the membranes (Saija et al., 1998). Quercetin, the most abundant molecule detected in all three extracts (present at amount ranging from 28.34 to 47.65 μg/g of EVOO, Table 2), exhibited a different behavior. It apparently was able to influence positively the antioxidant activity up to a concentration of 34.43 μg/g. On the other hand, at higher values, quercetin seemed to exert an inhibitory action on the antioxidant activity, which decreased from 34% to 20.7% (Figure 2c). A similar behavior could be also attributed to luteolin (Figure 2d), which amounts in the three extracts ranged between zero and 9.77 μg GAE/g. Like quercetin, it apparently exerted an antioxidant activity (34%) until a specific threshold (that could be ascribable to 5.09 μg GAE/g); after which, increasing its content until 9.77 μg GAE/g, the antioxidant activity decreased from 34% to 16%. Formononetin (corr = −37.98) showed a variable trend so that increasing its amount until a certain percentage (7.6%) the antioxidant activity decreased, increasing again as the molecule's content increased (Figure 2e). Our results corroborated the hypothesis that a certain bioactive compound can modify its properties in the presence of other compounds. In the case of spiraeoside, for instance, it is possible that its influence on the antioxidant activity can be negligible (Figure 2f), although its presence in the extracts of EVOOs *Ruvea antica* and *Ogliarola* was practically the same.

### 3.3 Volatile compounds analysis

The analysis of EVOO volatile compounds was performed through the SPME sampling followed by GC‐MS (Torri, Sinelli, & Limbo, 2010). SPME, as an alternative technique for fractionation of volatiles from interfering non‐volatile matrix compounds, is a pre‐concentration technology, which integrates sample extraction, concentration, and sample introduction into a single solvent‐free step, preventing the production of artifacts compared with conventional solvent extraction procedures (Pawliszyn, 2012). A total of 49 VOCs were identified, which belonged to hydrocarbons (3), aldehydes (11), alcohols (12), ketones (5), esters (5), carboxylic acids (6), terpenes (6), and others (1). Identification of volatile metabolites was carried out by (a) associating the GC retention time and mass spectra with those of pure commercial standards, when available, (b) matching MS spectra for each putative compound with those of NIST and Wiley libraries (https://chemdata.nist.gov/dokuwiki/doku.php?xml:id=chemdata:ridatabase; https://www.sisweb.com/software/wiley-registry.htm), and (c) comparing the Kovats indexes calculated by using a C8‐C40 *n*‐alkane series to literature data for similar chromatographic conditions. SPME GC‐MS semiquantitative data, calculated as the percent ratio of the respective peak area relative to the peak area of 4‐methyl‐2‐pentanol, used as internal standard, were subject to a one‐way ANOVA, in order to investigate the effect of cultivar on the identified VOCs. Table 3 reports the ANOVA of the 49 detected volatiles, the abbreviation code, the experimental and literature reported Kovats index, and the identification methods. Data reported in this table showed that the three cultivars share 30 common metabolites. Anyway, some VOCs can be considered cultivar‐specific flavor compounds, as they are present only in one cultivar. At this concern, heptane (H1), 2‐methyl butanal (Ald1), heptanal (Ald5), 1‐octanol (Al10), and 2‐methyl ethyl butanoate (E2) are found only in EVOO *Ogliarola*, while butanoic acid (A2) and α‐muurolene (T5) were exclusively present in EVOO *Ravece*. 1‐Butanol‐3‐methyl (Al2) was detected only in the VOCs profile of *Ruvea antica*. Aldehydes were the most abundant VOCs in *Ogliarola* and *Ravece* EVOOs, representing the 84.7% and 64.0% of total volatile compounds, respectively. Among aldehydes, (E) 2‐hexenal produced by the LOX pathway was notably predominant, representing a percentage of 79.8% of total volatiles in *Ogliarola* and 59.6% in *Ravece*. These results agree with previous studies reporting (E) 2‐hexenal among the principal volatiles normally found in VOCs of EVOOs produced in the Mediterranean basin (Salas et al., 2013). This compound provides the typical “green note” of olive oil and has been reported to be negatively correlated with the maturity and degree of oxidation of virgin olive oils (Pouliarekou et al., 2011). Alcohols were the most abundant volatiles present in the EVOO of *Ruvea antica*, representing the 58.2% of the total VOCs. The principal alcohols were (E) ‐hexen‐1‐ol (37.4%) and 1‐hexanol (15%), both deriving from the LOX pathway and showing a characteristic odor described as green, grassy, leafy. These compounds, on the other hand, were present only at lower concentration in *Ogliarola* (1.2% and 1.8%, respectively) and in *Ravece* EVOOs (4.2% and 14.1%, respectively).

**Table 3:** Volatile organic compounds (VOCs) in the three EVOOs

VOCs	Code	RIt/RIsp	ID	*Ogliarola*		*Ravece*		*Ruvea antica*		*p*
Hydrocarbons										
Heptane	H1	700/700	RI/MS/S	1.23	a	nd	b	nd	b	***
Octane	H2	800/800	RI/MS/S	3.28	a	2.11	ab	1.19	b	**
Toluene	H3	1021/1022	RI/MS/S	0.75	a	0.52	ab	0.33	b	*
Aldehydes										
Butanal 2‐methyl	Ald1	878/880	RI/MS/S	0.54	a	nd	b	nd	b	***
Hexanal	Ald2	1057/1058	RI/MS/S	10.38	a	9.72	a	3.97	b	*
2‐Pentenal (E)	Ald3	1111/1111	RI/MS/S	0.83	a	0.69	a	nd	b	***
3‐Hexenal (Z)	Ald4	1124/1126	RI/MS	1.53	a	1.98	a	nd	b	***
Heptanal	Ald5	1180/1182	RI/MS/S	0.36	a	nd	b	nd	b	***
2‐Hexenal (E)	Ald6	1213/1207	RI/MS/S	292.63	a	208.48	a	31.24	b	***
Octanal	Ald7	1292/1287	RI/MS/S	0.53		0.41		3.26		ns
2‐Heptenal (Z)	Ald8	1324/1320	RI/MS/S	0.61	a	0.28	ab	nd	b	***
2,4‐Hexadienal	Ald9	1394/1397	RI/MS	0.71	a	0.54	a	nd	b	***
2,4‐Heptadienal	Ald10	1465/1451	RI/MS	0.71	a	0.72	a	0.35	b	**
Nonanal	Ald11	1391/1385	RI/MS/S	1.64		1.16		11.62		ns
Alcohols										
1‐Penten‐3‐ol	Al1	1156/1157	RI/MS/S	2.75		3.46		2.18		ns
1‐Butanol‐3‐methyl	Al2	1211/1212	RI/MS/S	nd	b	nd	b	0.72	a	***
1‐Pentanol	Al3	1256/1255	RI/MS/S	nd	b	0.42	b	1.44	a	***
2‐Penten‐1‐ol (Z)	Al4	1319/1312	RI/MS/S	0.30		0.61		0.42		ns
2‐Penten‐1‐ol (E)	Al5	1326/1327	RI/MS	2.80	ab	4.56	a	1.79	b	**
1‐Hexanol	Al6	1358/1360	RI/MS/S	4.52	b	14.85	b	30.63	a	***
3‐Hexen‐1‐ol (E)	Al7	1368/1351	RI/MS/S	nd	b	0.43	a	0.40	a	**
3‐Hexen‐1‐ol (Z)	Al8	1385/1390	RI/MS/S	4.21	b	10.43	a	4.45	b	**
2‐Hexen‐1‐ol (E)	Al9	1405/1400	RI/MS/S	6.63	b	49.26	a	76.08	a	***
1‐Octanol	Al10	1562/1562	RI/MS/S	0.28	a	nd	b	nd	b	***
Benzyl alcohol	Al11	1866/1865	RI/MS/S	0.16		0.11		0.10		ns
Phenylethyl alcohol	Al12	1901/1859	RI/MS/S	0.69	a	0.34	b	0.21	b	***
Ketones										
3‐Pentanone	K1	984/984	RI/MS/S	2.27	b	6.36	b	20.51	a	***
2‐Pentanone‐4‐methyl	K2	1003/1006	RI/MS/S	9.99	ab	16.26	a	6.60	b	**
1‐Penten‐3‐one	K3	1012/1014	RI/MS/S	8.32	a	2.87	b	nd	b	***
2‐Octanone	K4	1286/1285	RI/MS/S	0.07		0.05		0.07		ns
6‐Methyl‐5‐hepten‐2‐one	K5	1338/1336	RI/MS/S	0.34		0.32		0.33		ns
Esters										
Ethyl acetate	E1	853/854	RI/MS/S	0.14	b	0.11	b	0.33	a	***
2‐Methyl ethyl butanoate	E2	1034/1033	RI/MS/S	0.16	a	nd	b	nd	b	***
Hexyl acetate	E3	1277/1307	RI/MS/S	0.24	a	0.16	a	nd	b	***
3‐Hexen‐1‐ol acetate (Z)	E4	1321/1390	RI/MS	0.57	b	2.47	a	0.93	b	**
Methyl salicylate	E5	1767/1760	RI/MS/S	0.34		0.34		0.23	ns	
Carboxylic acids										
Propanoic acid	A1	1543/1523	RI/MS/S	0.53		0.41		0.34		ns
Butanoic acid	A2	1633/1619	RI/MS/S	nd	b	0.13	a	nd	b	***
Hexanoic acid	A3	1851/1850	RI/MS/S	0.76		0.72		0.61		ns
Heptanoic acid	A4	1918/1913	RI/MS/S	0.44	ab	0.77	a	nd	b	**
Octanoic acid	A5	2048/2046	RI/MS/S	1.57		2.72		1.14		ns
Nonanoic acid	A6	2174/2171	RI/MS/S	0.90	ab	1.42	a	0.12	b	*
Terpenes										
dl‐Limonene	T1	1183/1185	RI/MS/S	0.29	a	nd	b	0.18	a	**
cis β‐Ocimene	T2	1252/1245	RI/MS/S	0.46	b	0.93	a	0.44	b	*
α‐Copaene	T3	1481/1488	RI/MS	0.21	b	1.72	a	0.39	b	***
α‐Bergamotene	T4	1579/1580	RI/MS	nd	b	0.19	a	0.12	ab	**
α‐Muurolene	T5	1721/1722	RI/MS	nd	b	0.22	a	nd	b	***
α‐Farnesene	T6	1721/1751	RI/MS/S	0.57	a	nd	b	0.18	b	***
Others										
Aniline	O1	1753/1754	RI/MS/S	0.49		0.73		0.64		ns

Note

Data are the mean values of three samples. For each metabolite, the mean values followed by a different letters are significantly different (*p* < .05) according to one‐way ANOVA test (ns = not significant; *, **, *** = significant at values < .05, .01, .001). Concentrations were calculated as RPA (%). ID = Identification method as indicated by the following: RI: Kovats retention index on the HP‐Innowax column; MS: NIST and Wiley libraries spectra; S: co‐injection with authentic standard compounds on the HP‐Innowax column. RIt/RIsp: Relative theoretical retention index versus Relative retention index experimentally calculated against n‐alkanes (C8‐C20) on the HP‐Innowax column.

**Figure 3:**
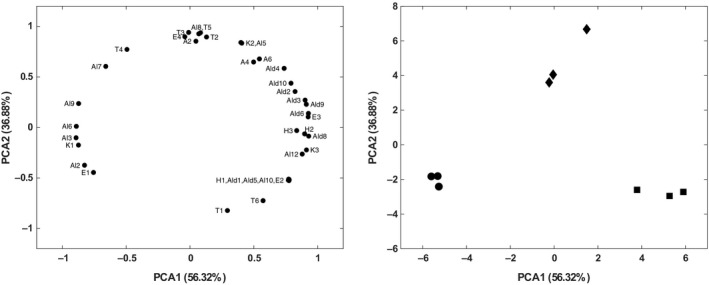
2D‐PCA plots of the VOCs profiles of the three EVOOs. The loadings (left) and the three (scaled) samples of *Ogliarola* (■), *Ravece* (♦), and *Ruvea antica* (●) in the transformed space (right)

The variety could strongly affect the abundance of volatile compounds, which in turn have revealed to be extremely valuable as varietal markers (Kalua et al., 2005). For this reason, the volatile profiles of the three EVOOs were subjected to multivariate statistical analysis with the aim to build models able to explain the variations of the metabolic content dependently from genotype, and to identify putative volatile markers useful for cultivar discrimination. Exploratory data analysis performed by PCA was applied to evaluate the effectiveness of VOCs profiles on the cultivar distinction. The cross‐validation test showed that two principal components were required to explain the total variability of analyzed parameters. The eigenvalues of the covariance matrix demonstrated that the two principal components (PCs) accounted for more than the 93% of the total explained variance. Principal component analysis model generated the 2D‐plots presented in Figure 3, where the presence of three distinctive clusters corresponding to the different cultivars is highlighted. This result confirms that, considering the volatile profile, the variety dominated over other factors (geography, maturity stage, and quality) in distinction of EVOOs of different ecotypes, even when, as in our case, produced in a very small area and with the same agriculture practices and postharvest handling (Lin et al., 2013; Zhang et al., 2010). Moreover, the ReliefF algorithm, a filter‐based method for feature selection, showed that the four most important potential marker volatiles responsible for the discrimination of the three olive oil cultivars are 1‐hexenol (Al6), 2‐hexen‐1‐ol (Al9), 3‐pentanone (K1), and 2‐hexenal (Ald6). These findings are consistent with PCA data (Figure 3), according to the fact that Al6, Al9, and K1 are the most abundant volatiles in EVOO *Ruvea antica*, while Ald6 is the main compound both in the EVOOs *Ogliarola* and *Ravece*.

## 4 CONCLUSION

Data obtained in this research clearly confirm the influence of genetic and environmental factors in determining the organoleptic properties of olive oil and permitted a distinction of the three EVOOs studied on the basis of their volatile constituents.

## CONFLICT OF INTEREST

Authors declare that they do not have any conflict of interests.

## ETHICAL APPROVAL

Human and animal testing was unnecessary for this study.
